# Global assistance in caring for Syrian refugees

**DOI:** 10.1186/s13031-016-0074-9

**Published:** 2016-03-23

**Authors:** Mujalli Mhailan Murshidi

**Affiliations:** University of Jordan, Amman, Jordan

**Keywords:** Syrian refugees, Neonates

## Abstract

ᅟ

## Main text

In 2013, while I was the Minister of Health and Minister of Environment in Jordan, the majority of the neonates at neonate unit at the Mafraq Maternity and Neonate Hospital in northern Jordan were borne to Syrian women (Syrian refugees needed up to 18 of the 20 beds most of the time during that year). This makes me agree with the work of Fuat Emre Canpolat et al. which concluded that further research is needed to understand the relative morbidity of babies born to Syrian refugees compared to the local population, as well as the economic impact on facilities treating these cases [1]. Jordan is committed to the health of both Syrians and Jordanians. However, the health system is dangerously overstretched. Excessive demands on the health system pose risks to the health status and social stability. The international community is invited to better assist in the efforts to fulfil these demands [2].

A recent editorial in *The Lancet* stated clearly that the WHO’s European Health Report 2015 lacks attention, and strikingly so, to preparedness and provision for the health of refugees and migrants [3]. The ongoing large-scale movement of people escaping conflict in Syria and other countries has had a dramatic effect on several European region states, with two million or more people having entered Turkey and migration to other European countries ongoing. It is, at the very least, disappointing that a flagship WHO Europe health report should overlook such a major challenge to comprehensive and equitable health provision in the region. The next report should be holistic in specifically addressing planning and provision for migrants’ health in all of the region's affected countries [3]. Finally, it should be stressed that WHO and UN Organizations reports bring attention to preparedness and the provision of health care for refugees and migrants, in Europe, Jordan and in other areas affected by this regional crisis, for better planning and active support.

## Ethics approval and consent to participants

Not applicable.

## Consent for publication

Not applicable.

## Availability of data and material

Available.
